# Stem cell therapy for amyotrophic lateral sclerosis

**DOI:** 10.1186/s13619-015-0026-7

**Published:** 2015-11-19

**Authors:** Zhijuan Mao, Suming Zhang, Hong Chen

**Affiliations:** 1 Department of Neurology of Tongji Hospital, Tongji Medical College, Huazhong University of Science and Technology, Wuhan, China; 2 Department of Rehabilitation of Tongji Hospital, Tongji Medical College, Huazhong University of Science and Technology, Wuhan, China

**Keywords:** Amyotrophic lateral sclerosis, Stem cells, Cell transplantation, Clinical translation

## Abstract

Amyotrophic lateral sclerosis (ALS) is a fatal neurodegenerative disorder characterized by the loss of motor neurons. Currently, no effective therapy is available to treat ALS, except for Riluzole, which has only limited clinical benefits. Stem-cell-based therapy has been intensively and extensively studied as a potential novel treatment strategy for ALS and has been shown to be effective, at least to some extent. In this article, we will review the current state of research on the use of stem cell therapy in the treatment of ALS and discuss the most promising stem cells for the treatment of ALS.

## Introduction

Amyotrophic lateral sclerosis (ALS) (or Lou-Gehrig’s disease) represents a neurodegenerative disorder characterized by progressive degeneration of motor neurons and its symptoms including muscle atrophy, weakness, fasciculation, and spasticity [1]. The condition is the most common motor neuron disease, with a worldwide incidence of 2–3 per 100,000 and a prevalence at 4–6 per 100,000 [2], posing a heavy burden on both the families involved and society at large. Patients tend to die 3–5 years after diagnosis due to progressive motor neuron loss and weakness of skeletal muscles, especially those muscles responsible for breathing, which is the primary cause of death caused by ALS [3]. The pathogenesis of ALS is believed to be multifactorial. For the familial forms, several genetic mutations have been identified as being associated with the disease, including mutations in Cu superoxide dismutase (SOD1), TAR DNA binding protein-43 (TDP-43), the C9orf72 gene (the most common mutation underlying familial forms of ALS), and the recently discovered TBK1 gene encoding a protein involved in two essential cellular pathways of emerging interest in ALS research: autophagy and inflammation [4]. In the more common forms of sporadic ALS, neurodegeneration might result from an intricate interaction among multiple cell types and several different mechanisms, including protein aggregation, glutamate-mediated excitotoxicity, mitochondrial dysfunction, oxidative stress, impaired axonal transportation, altered glial cell function, and deficiency of neurotrophic factors [5]. All of these factors can eventually lead to the disruption of axonal transport processes via intracellular accumulation of neurofilaments [3, 6]. This heterogeneity of ALS makes it difficult to identify the exact cause of ALS and so develop effective therapies. Except Riluzole, which is believed to be able to extend survival by a few months [7], to date, few treatments have proved to be highly or consistently effective [8].

Stem cell therapy is a promising potential treatment option for ALS, given stem cells’ remarkable plasticity and ability to differentiate into multiple neuronal lineages [9]; they are consequently a valuable source for replacement cell therapy. When locally or systemically transplanted, stem cells are capable of migrating to disease-associated loci to exert the desired therapeutic effect [10]. Currently available cell therapies may take advantage of a variety of stem cells to modify disease pathophysiology [11], slow down or even halt the progression of disease, possibly by providing protective factors to surrounding cells, modulating the host immune environment, inhibiting inflammation, or even replacing injured cells [12–17]. Several types of stem cells have been studied as possibilities for treating ALS, including neural stem cells (NSCs), mesenchymal stem cells (MSCs), glial-restricted progenitor cells (GRPs), embryonic stem cells (ESCs), and induced pluripotent stem cells (IPSCs) [18]. Here, we will comprehensively review the current state of research concerning treatments for ALS using stem cells and provide information on aspects of further research into stem cell-based therapies for ALS.

### Neural stem cells

NSCs originate from the neuroectoderm of early embryos and are found in embryonic, fetal, and adult nervous systems. They possess the potential to differentiate into any cell type in the central nervous system (CNS) (although NSCs derived from adult tissues show a more limited differentiation capacity [19]). The integration ability and prospective therapeutic efficacy of human neural stem cells (hNSC) has been demonstrated in rodent models of neurological diseases [20–23]. Apart from regenerating lost neuronal cells, NSCs can also improve the functional outcomes of rats through auxiliary mechanisms, such as neurotrophism [24–26] and immunosuppression [27–29].

A number of studies have demonstrated that NSC therapy had beneficial effects on ALS rats [17, 30]. Transplanted NSCs could differentiate into neurons and form synaptic connections with host tissues, delay disease onset and progression, and prolong the survival of experimental animals [17]. Hefferan et al. found that grafted hNSCs protected adjacent motor neurons and helped to achieve transient functional improvement [31], and they speculated that this transient functional improvement was attained possibly because transplanted NSCs elicited neurogenesis and triggered intrinsic repair mechanisms in the spinal cord [32]. More encouragingly, Teng and co-workers found that besides a delay in disease progression and an improvement in motor function, a quarter of the NSC-grafted ALS mice survived three times longer than their non-grafted counterparts [33].

Given the pre-clinical support for NSC-based therapies, in 2009, the FDA approved a clinical trial on the safety and tolerability of surgical delivery of stem cells and any resulting cell toxicity [34]. A total of 18 patients with ALS received an intraspinal fetal-derived NSC (NSI-566RSC) engraftment following a risk escalation paradigm, progressing from non-ambulatory to ambulatory subjects, lumbar to cervical spinal cord segments, and unilateral to bilateral injections across five cohorts. After monitoring the patients for 2.5 years, all patients tolerated the procedure without major surgical complications, such as injection-attributable neurological worsening, and there were no indications that the stem cells themselves were either toxic or injurious to the spinal cord. In an expansion of the above study using NSCs isolated from human fetal spinal cord tissues, Mazzini et al. transplanted human fetal brain tissues into the anterior horns of the spinal cord and additionally used a much higher cell dosage and a milder immunosuppression regimen [35]. They verified the safety and tolerability by clinical assessment against safety measures and follow-up, utilizing neuroimaging and other techniques [35]. These studies have paved the way for future clinical trials on the efficacy and dosage of NSC treatment for ALS. A phase I clinical trial that began in July 2011 is designed to verify the safety of expanded hNSCs and microsurgery and to evaluate their effect on the quality of life of the patients (ClinicalTrials.gov Identifier: NCT01640067). A phase II clinical trial, which started in May 2013, is aiming to assess the feasibility, safety, toxicity, and maximum tolerated (safe) dose of the NSC treatment (ClinicalTrials.gov Identifier: NCT01730716).

However, in addition to two issues which hamper NSC studies, namely ethical issues and immune rejection problems, NSCs are derived from fetal spinal cord (NSI-566RSC) [36] or fetal brain tissues [35], two sources of cells that are very limited. Consequently, their large-scale use in clinical trials remains a challenge.

### Mesenchymal stromal cells

MSCs are multipotent adult stem cells that can be easily extracted from various adult connective tissues (i.e., bone marrow and adipose tissue) and can differentiate into a variety of cells [37–39].

A number of studies employing animal models of ALS have investigated the therapeutic potential of MSCs by injecting cells either peripherally or directly into the spinal cord. Marconi et al. assessed the efficacy of the systemic administration of adipose-derived mesenchymal stem cells (ASC) in SOD1-mutant mice and found that the cells not only significantly delayed motor deterioration for 4–6 weeks and maintained the number of motor neurons but also up-regulated the levels of glial-derived neurotrophic factor (GDNF) and basic fibroblast growth factor (bFGF) in the spinal cord. Given that ASCs produce bFGF but not GDNF, these findings indicated that ASCs may promote neuroprotection either directly and/or by modulating the response of local glial cells toward a neuroprotective phenotype [40]. Similarly, intramuscular transplantation of MSCs engineered to secrete GDNF was found to attenuate motor neuron loss and prolong the lifespan of ALS rats [41]. In another study, MSCs were genetically modified to release GDNF or VEGF, and when injected into animals, they extended survival and alleviated the loss of motor function [42]. The therapeutic effect of MSCs may primarily capitalize on innate trophic support from themselves or from the delivery of augmented growth factors. Intraspinal, intracerebral, intrathecal, and intravenous injection of autologous MSCs in SOD1-G93A mice have also reported beneficial effects on disease progression, including slowed loss of motor neurons, improved motor function, and extended survival [43–48]. Given the fact that MSCs can deliver neurotrophic, anti-inflammatory, and immunomodulatory molecules [49, 50], these cells are a promising treatment approach for ALS.

In 2003, Mazzini and colleagues conducted the first clinical studies to determine the safety and tolerability of direct intraparenchymal transplantation of MSCs for the treatment of ALS. MSCs were isolated from allogeneic ALS patients’ bone marrow aspirates and transplanted into the thoracic spinal cord. While there was no functional improvement following MSC transplantation, no serious adverse effects and no detrimental effects on neurological function were reported [51]. Their follow-up studies, lasting more than 4 years after surgery, revealed no signs of toxicity or abnormal cell growth and showed that four patients might have benefited from the treatment [52, 53]. Subsequently, a number of clinical trials have evaluated autologous MSC transplantation and demonstrated that intraspinal, intrathecal, and intracerebral transplants were safe and feasible [54]. It is worth mentioning that three clinical studies innovatively mobilized endogenous MSCs by using granulocyte-colony stimulating factor (G-CSF) in ALS patients, and their MSCs were instantaneously increased and no major adverse events were found in any of the three studies [55–57]. Moreover, BrainStorm Cell Therapeutics developed a cell type trademarked as “NurOwn™” for the treatment of ALS. The cells can differentiate into specialized neuron-supporting cells capable of stably secreting neurotrophic factors (MSC-NTFs). Currently, a phase II clinical trial using NurOwn cells began in 2014 to evaluate the safety and efficacy of the cells (ClinicalTrials.gov Identifier: NCT02017912). Another phase II study using NurOwn cells, this time in a dose-escalating clinical study, is now under way (ClinicalTrials.gov Identifier: NCT01777646).

MSCs can be relatively easily obtained from adult tissues, and their application does not pose substantial ethical issues [58, 59], and because ALS does not influence MSC expansion and differentiation [60], the cells can be extracted from patients themselves, thus avoiding immune rejection. So, MSCs seem to be an attractive candidate for ALS cell therapy. However, deriving MSCs from either bone marrow or adipose tissue causes, to some degree, trauma. What is more, MSCs are of mesodermal origin and thus their ability to transdifferentiate into neuronal cells of ectodermal origin is questionable [61]. And as far as we know, so far, there are no robust pre-clinical studies on the long-term safety, in vivo differentiation, dosage, and biological activity of human MSCs used for the treatment of ALS. Therefore, studies on its further application in clinical practice are warranted.

### Glial-restricted progenitor transplantation

Many studies have looked into the role of astrocytes in ALS and demonstrated that astrocytes derived from SOD1 mice and ALS patients could induce motor neuron death, possibly through a Bax-dependent mechanism triggered by toxic soluble factors (termed “gliotransmitters”) [62–66]. When astrocytes derived from SOD1 glial progenitors were transplanted into mice, they could induce host motor neuron death, focal weakness of the corresponding limb, and gliosis of host astrocytes and microglia [67, 68]. Recent research has demonstrated that human glial progenitor transplantation and gene expression was independent of the ALS neurodegenerative spinal cord environment [69], indicating that some cell autonomous changes take place in astrocytes expressing ALS-linked mutations and treatment of ALS with astrocytes is feasible.

GRPs are the earliest progenitor cell type derived from the embryonic spinal cord, and they show a tripotential phenotype in their ability to differentiate to oligodendrocytes and two types of astrocytes [70, 71]. Lepore et al. isolated GRPs from the rat embryonic spinal cord and transplanted them into the cervical spinal cord of SOD1-G93A rats. After injection, they found that the grafted cells survived in the diseased tissues and differentiated efficiently into astrocytes, with microgliosis alleviated at the transplanted sites; additionally, survival was extended, motor neuron loss was ameliorated and declines in forelimb motor capability slowed, and respiratory functions improved [72]. Later, another study conducted by the same research team showed that hGRP (also referred to as Q-Therapeutics’ Q-Cells®) derived from human fetal forebrain robustly survived and migrated into both gray and white matter and differentiated into astrocytes in SOD1-G93A mice spinal cord. However, Q-Cells engraftment did not lead to motor neuron protection or any therapeutic benefits in terms of functional outcome measures [73]. The discrepancies between the two GRP transplantation studies may be due to the differences in cell types (allograft versus xenograft, rat spinal versus human forebrain-derived), cell maturity, the number of injection sites, and transplanted cells [74]. As the functions of astrocytes vary depending upon their origins [75], the conflicting results suggest to us that further research should be conducted to understand the influence of cell type and cell number on clinical outcome.

Like hNSCs, hGRPs are derived from fetal forebrain tissues [76], and their widespread clinical application is greatly hampered by the scarcity of resources, ethical issues, and potential for immune rejection.

### Embryonic stem cells

ESCs, derived from the inner cell mass of the blastocyst [77], can be efficiently differentiated into any cell type both in vitro and in vivo, and these differentiated cells present morphological, biochemical, and physiological traits similar to their in vivo counterparts. Many important and decisive differentiation factors have been discovered, and simple protocols for ESC differentiation into motor neurons are available.

In 2005, Zhang et al., for the first time, reported the successful differentiation of human ESC (hESC) into a motor neuron (MN) phenotype [78]. Wyatt et al. transplanted hESCs-derived motor neuron progenitors (hMNPs) into three animal models of motor neuron loss: SMA (Δ7SMN), ALS (SOD1-G93A), and spinal cord injury (SCI) [79]. The transplanted cells survived, differentiated, and secreted physiologically active growth factors in vivo, thereby significantly increasing the number of spared endogenous neurons. The ability to maintain dying motor neurons by providing motor neuron-specific neurotrophic support is a powerful potential treatment strategy for ALS.

Though the use of hESCs in treating ALS in animals are encouraging, not all hESC lines can differentiate into neural lineages, probably because of inherent differences and/or the underlying genetics of the embryos from which the lines were derived [80, 81]. In addition, hESCs are derived from pre-implantation human embryos [82], and although hESCs can be maintained and expanded indefinitely, their use comes with significant ethical concerns and potential immune response issues.

### Induced pluripotent stem cells

IPSCs can be derived from patients’ somatic cells by reprogramming with specific factors [83]. iPSCs express stem cell markers and have the ability to give rise to all three germ layers, as these cells are derived from adult somatic tissues they bypass ethical concerns, and so are promising candidates for stem cell therapy for ALS.

In 2008, Dimos et al. developed the first strain of human iPSCs from an 82-year-old familial ALS woman [84]. Mitne-Neto and colleagues successfully reprogrammed fibroblasts from an ALS8 patient into pluripotent stem cells and differentiated them into motor neurons [85]. Popescu et al. showed that iPSC-derived neural progenitors efficiently engrafted into the adult spinal cord and could survive in high numbers [86]. They postulated that the transplantation of stem cell-derived neural progenitors might exert a dual beneficial effect: replacing lost motor neurons and serving as a source of neurotrophic factors and modifiers of the toxic environment. Recently, a similar study generated and purified a specific NSC population from human iPSCs. After injection of these cells into SOD1-mutated mice, NSCs engrafted and migrated into the CNS, resulting in improved neuromuscular function and motor unit pathology and a significantly prolonged life span [87]. These beneficial effects are believed to be linked to multiple mechanisms, including production of neurotrophic factors and reduction of microgliosis and macrogliosis, thus leading to increased resistance to death of motor neurons and neurodegeneration. In a series of long-term studies, Chen et al. showed iPSC-derived neural progenitors mostly differentiated into astrocytes, replaced the endogenous astrocytes, formed networks through their processes, and encircled endogenous neurons [68]. Preclinical studies, currently being conducted at Johns Hopkins University, use human iPSC-derived glial-restricted precursors (iPSC-GRPs) [88] and may offer perspectives for the use of iPSC-based therapy in ALS [87].

Among all the cells mentioned above, hiPSCs have incomparable advantages over other cells. Currently, hiPSCs can be obtained from the blood [89] or urine [90] and so are relatively easily available, rendering their use feasible in clinical treatment. Therefore, hiPSCs are an attractive candidate for ALS cell therapy. A major concern about the application of hiPSCs might be its potentially high tumor-forming capability.  However, in actual research of hiPSCs, tumor formation was rare [68, 86, 91, 92], except for a very small proportion of grafts that remained positive for neural progenitor marker genes [91]. If the number and the differentiation of transplanted neural progenitor cells are well controlled, the possibility for tumor formation can be substantially decreased. Additionally, concerns about random viral integration could be ameliorated by the development and optimization of the use of episomal plasmids, recombinant proteins with membrane permeable peptides, or Sendai virus vectors.

## Conclusion

Riluzole, the only FDA-approved treatment for ALS, has only a slight positive effect on survival and function in some patients and poses a high financial burden on patients and their families. Over the past 20 years, the results of most clinical trials on other drugs have been disappointing [93], making it urgent to find new and effective alternatives for the treatment of ALS. With the advent of regenerative medicine, stem cell transplantation has emerged as a promising potential new avenue of treatment for a wide array of degenerative diseases for which no specific or effective treatment is currently available.

Stem cell therapies may modify disease pathophysiology [11], slow down or halt the progression of disease, and even improve neuromuscular function and motor unit pathology, possibly by providing protective factors to surrounding cells, modulating the host immune environment, inhibiting inflammation, or even replacing injured cells [12–17]. For patients carrying genetic mutations related to ALS, genetic corrected stem cells could be generated to correct the mutation, and for those carrying no genetic mutation, the protective and replacing effect of allogeneic stem cells are available. The use of stem cells may modify disease physiology, decreasing protein aggregation, and reducing glutamate excitoxicity. The encouraging results of experimental studies on stem cell therapy may promote its clinical application for the treatment of ALS. Yet, the prospect of its clinical use depends on the resolution of some crucial issues. The most important concern that remains to be addressed is the uniform generation and preparation of the cells under good manufacturing practice (GMP).

For clinical application, cell replacement therapy needs to be thoroughly tested by standardized studies in vitro followed by rigorous clinical trials in humans. Although we still have a long way to go, the treatment of ALS with human stem cells remains a viable and promising alternative.

## Abbreviations

ALS: amyotrophic lateral sclerosis
ASC: adipose-derived mesenchymal stem cells
bFGF: basic fibroblast growth factor
CNS: central nervous system
ESC: embryonic stem cell
G-CSF: granulocyte-colony stimulating factor
GDNF: glial-derived neurotrophic factor
GRP: glial-restricted progenitor cell
IPSC: induced pluripotent stem cell
MN: motor neuron
MSC: mesenchymal stem cell
NSC: neural stem cell
SCI: spinal cord injury
SMA: spinal muscular atrophy
SOD1: superoxide dismutase 1
VEGF: vascular endothelial growth factor
